# Immediate Repair for Laceration of the Tunica Albuginea of the Corpora Cavernosa and Penile Urethral Injury Caused by Blunt Trauma to the Flaccid Penis: A Case Report

**DOI:** 10.1002/iju5.70173

**Published:** 2026-04-05

**Authors:** Rikushi Fujimura, Yoshiyuki Ishiura, Daimon Kurauchi, Takafumi Shimada, Renato Naito, Hiroaki Iwamoto, Susumu Niikura, Atsushi Mizokami

**Affiliations:** ^1^ Department of Integrative Cancer Therapy and Urology, Graduate School of Medical Science Kanazawa University Kanazawa Ishikawa Japan; ^2^ Department of Urology Toyama Rosai Hospital Uozu Toyama Japan; ^3^ Department of Urology Kaga Medical Center Kaga Ishikawa Japan

**Keywords:** blunt penile trauma, penile fracture, surgical repair, urethral injury

## Abstract

**Introduction:**

Blunt trauma to the flaccid penis rarely causes laceration of the tunica albuginea of the corpora cavernosa.

**Case Presentation:**

A 31‐year‐old male patient sustained blunt trauma to the flaccid penis during a basketball game, causing subcutaneous hematoma, hematuria, and urinary retention. Computed tomography demonstrated a laceration of the tunica albuginea of the corpora cavernosa. Immediate surgical exploration confirmed the laceration of the tunica albuginea and the partial disruption of the penile urethra. Both injuries were repaired, and a suprapubic catheter was inserted. On postoperative day 12, retrograde urethrography revealed no leakage; however, on postoperative day 24, leakage was observed at the injury site. The suprapubic catheter was maintained for 3 months. Final imaging confirmed urethral patency without leakage.

**Conclusions:**

We report a rare case of blunt trauma to the flaccid penis. Immediate repair was successful, and the patient recovered without stricture, although a compressive mechanism caused delayed urethral leakage.

## Keynote Message

Blunt trauma to the flaccid penis rarely causes laceration of the tunica albuginea of the corpora cavernosa. We report a rare case of laceration of the tunica albuginea of the corpora cavernosa with urethral injury caused by such trauma. Immediate surgical repair was performed, leading to a favorable outcome.

## 1 Introduction

Penile fracture is defined as rupture of the tunica albuginea of the corpora cavernosa caused by blunt trauma to the erect penis, whereas such trauma to the flaccid penis rarely causes this type of injury [1].

Urethral injury occurs in 1%–38% of penile fracture cases, with incidence varying based on geographic region and etiology [2, 3, 4]. Early surgical repair for penile fracture has been reported to lead to better outcomes with low complication levels [4, 5, 6, 7].

We report a rare case of immediate repair for blunt trauma to the flaccid penis that led to laceration of the tunica albuginea of the corpora cavernosa and partial disruption of the penile urethra.

## 2 Case Presentation

**FIGURE 1:**
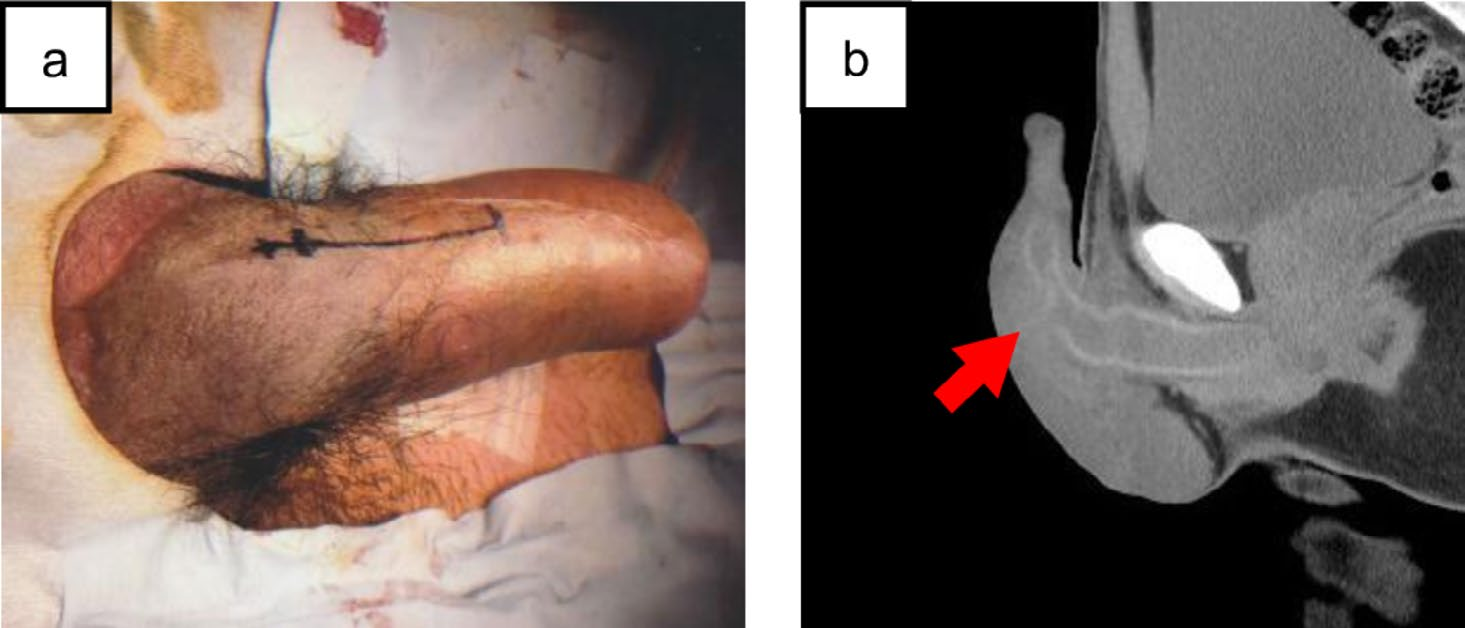
(a) Preoperative image of the penis with subcutaneous hematoma. (b) Computed tomography (CT) image demonstrating a laceration of the cavernosal tunica albuginea.

**FIGURE 2:**
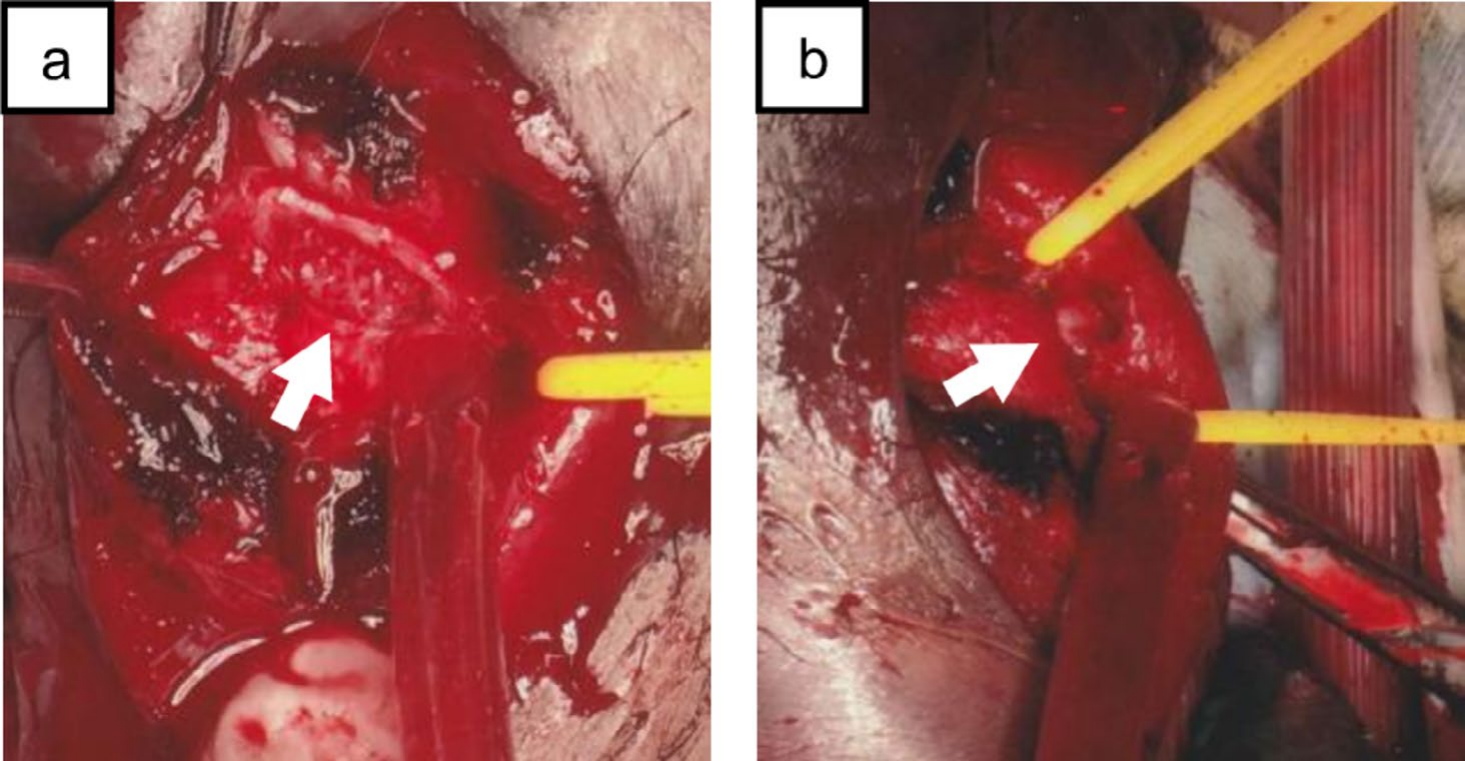
Intraoperative findings. (a) Laceration of the cavernosal tunica albuginea. (b) Partial disruption of the penile urethra extending over 180° on its dorsal aspect.

A 31‐year‐old male patient presented to our hospital the day after sustaining a blunt penile injury during a basketball game, when he was accidentally struck in the penis by an opponent's knee. Physical examination revealed penile pain, subcutaneous hematoma, hematuria, and urinary retention (Figure 1a). A computed tomography scan demonstrated a laceration of the tunica albuginea of the corpora cavernosa (Figure 1b). Immediate surgical exploration was performed to assess and repair the injury, which revealed a laceration of the tunica and a partial disruption of the penile urethra extending over 180° on its dorsal aspect (Figure 2a,b). Both injuries were repaired, and suprapubic and urethral catheters were inserted.

**FIGURE 3:**
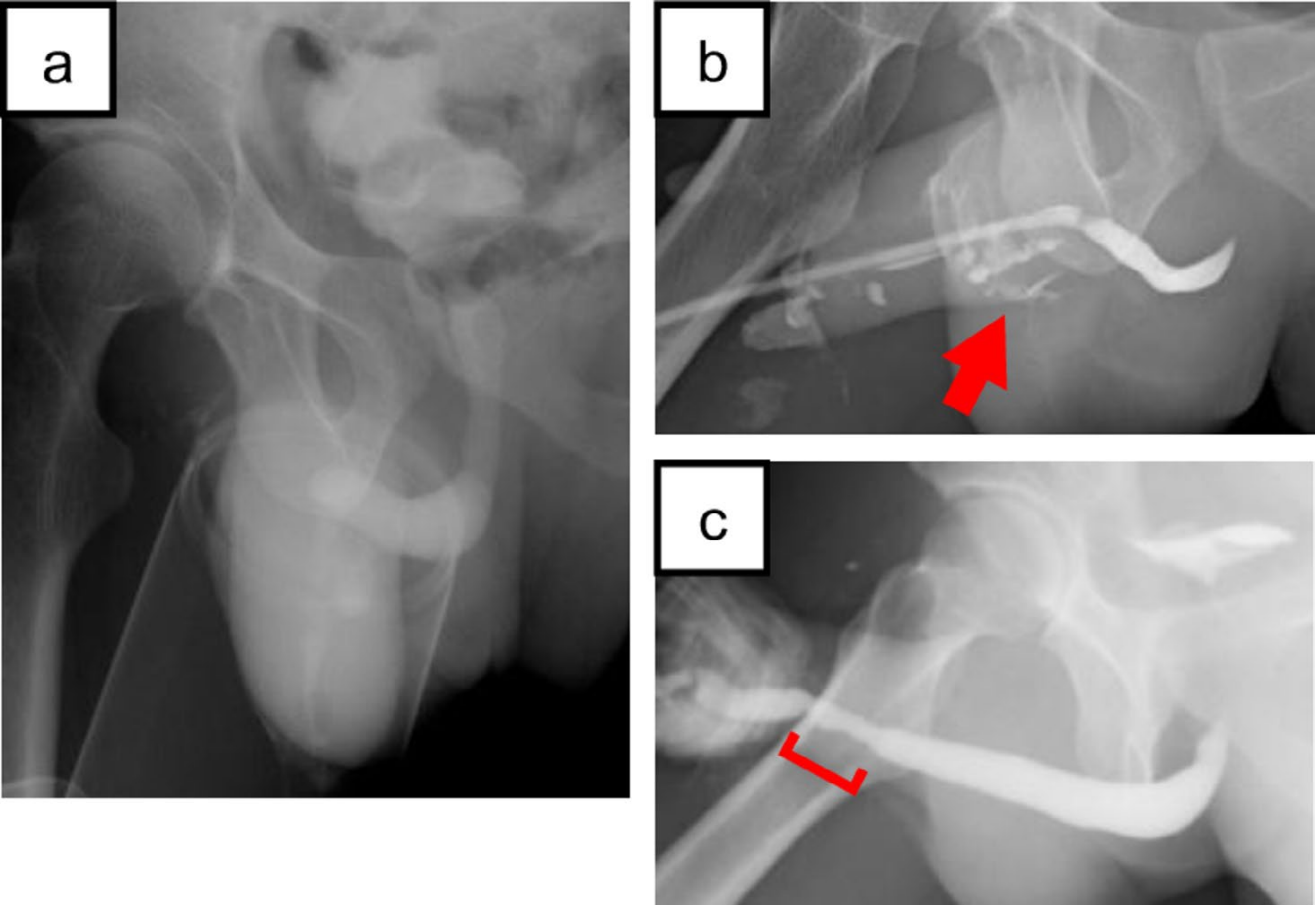
Retrograde urethrography (RUG) findings. (a) Postoperative day 12: No urethral extravasation. (b) Postoperative day 24: Urethral extravasation from the suture site on the ventral aspect of the penile urethra. (c) Postoperative day 48: No urethral extravasation, and a penile urethral stricture was suspected.

On postoperative day 12, retrograde urethrography (RUG) revealed no urethral extravasation (Figure 3a), and the urethral catheter was removed. On postoperative day 24, the patient presented with urinary leakage from the ventral aspect of the penis. RUG demonstrated urethral extravasation from the suture site on the ventral aspect of the penile urethra (Figure 3b). Suprapubic catheter drainage was then continued. On postoperative day 48, RUG indicated no urethral extravasation, although a urethral stricture in the penile urethra was suspected (Figure 3c).

**FIGURE 4:**
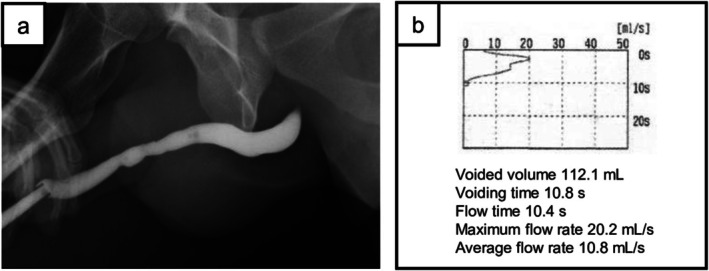
Three months postoperatively, (a) RUG revealed no urethral extravasation and urethral stricture resolution. (b) Uroflowmetry (UFM) demonstrated a normal urinary flow pattern without abnormalities.

Three months postoperatively, RUG revealed no urethral extravasation or urethral stricture (Figure 4a). The suprapubic catheter was then removed, and the patient subsequently reported no urinary symptoms. Uroflowmetry demonstrated a normal flow pattern (Figure 4b). Urethroplasty had been considered in a confirmed persistent urethral stricture, but clinical improvement during follow‐up rendered it unnecessary. Erectile function did not improve immediately after suprapubic catheter removal. Continued follow‐up would have been necessary to assess delayed erectile function recovery and to monitor urethral stricture development; however, the patient was lost to follow‐up.

## 3 Discussion

Blunt trauma to the flaccid penis, which causes laceration of the tunica albuginea of the corpora cavernosa with concomitant urethral injury, is an extremely rare traumatic condition, with only a few cases reported [8, 9, 10]. The tunica albuginea is approximately 2 mm thick in the flaccid penis, but it decreases to 0.25–0.5 mm during erection, making it more vulnerable to blunt external trauma during sexual activity or forced flexion [1, 5, 11]. In contrast, blunt external trauma to the flaccid penis is rare due to its mobility [8]. This case indicates the importance of considering tunical or urethral injuries even after blunt trauma to the flaccid penis.

Immediate surgical repair is widely acknowledged as the standard treatment for penile fracture, with or without urethral injury, as it is associated with favorable outcomes and low complication rates compared with delayed surgery or conservative management [2, 4, 5, 6, 7]. The Japanese Urological Association (JUA) guideline recommends performing immediate repair within 24 h when no urethral injury is present and as early as possible when urethral injury is concomitant, and notes that surgical repair is associated with fewer complications than conservative management [12]. However, temporary urethral extravasation occurred on postoperative day 24 in our case. Moreover, previous reports of flaccid penile injuries have not consistently demonstrated favorable courses [8, 9].

A typical penile fracture is not a compression‐type blunt injury [1]. In contrast, a straddle‐type injury causes bulbar urethral injury through compression between an external force and the pubic bone [13]. During immediate surgery, assessing the full extent of compressive damage can be challenging, and delayed tissue necrosis or urethral scarring leading to stricture may occur [8]. Accordingly, a staged strategy is commonly recommended: suprapubic urinary diversion with approximately three months of urethral rest, followed by delayed reassessment and reconstruction [1, 12, 13]. The mechanism is likely compressive in blunt trauma to the flaccid penis, similar to straddle‐type injury, and distinct from typical penile fracture. Thus, close follow‐up is required to detect complications of urethral contusion [8].

In compression‐type blunt injuries, the JUA guideline recommends a staged strategy with initial urinary diversion and delayed reassessment/reconstruction [12]. However, the evidence comparing delayed and immediate repair in blunt anterior urethral injuries is limited, with no clear superiority of either approach. The European Association of Urology (EAU) guideline states that immediate anterior urethroplasty in blunt injuries is highly controversial and should only be performed by a dedicated urethral surgeon.

In our case, a laceration of the tunica albuginea was present; therefore, this injury was managed according to the penile fracture treatment algorithm. Immediate surgery was performed under the supervision of an experienced urologist with expertise in urethral trauma, consistent with the EAU guideline's recommendation.

Immediate repair should be considered for blunt trauma to the flaccid penis when tunical laceration is suspected. As noted in the EAU guideline, compression‐type urethral injuries should be managed by a dedicated urethral surgeon if immediate repair is performed. Clinicians should remain alert to possible urethral contusion in the early postoperative period, even after immediate surgical repair, when a compressive mechanism is suspected.

## 4 Conclusion

We report a rare case of blunt trauma to the flaccid penis causing laceration of the tunica albuginea of the corpora cavernosa and the partial disruption of the penile urethra. Immediate surgical repair resulted in a favorable outcome. In blunt trauma to the flaccid penis, immediate repair should be considered when tunical laceration is suspected, following penile fracture management principles. Furthermore, when compression‐type urethral injury is a concern, surgery ideally should be managed by a dedicated urethral surgeon. Close follow‐up is essential, as delayed complications, including urethral stricture, may occur even after primary repair [13].

## Ethics Statement

The authors have nothing to report.

## Consent

The authors have nothing to report.

## Conflicts of Interest

The authors declare no conflicts of interest.

## Data Availability

Research data are not shared.
